# Effect of an interdisciplinary intervention with motivational approach on exercise capacity in obese adolescents: a randomized controlled clinical trial

**DOI:** 10.31744/einstein_journal/2020AO5268

**Published:** 2020-05-13

**Authors:** Letiane Bueno Zanatta, João Paulo Heinzmann-Filho, Fernanda Maria Vendrusculo, Natália Evangelista Campos, Margareth da Silva Oliveira, Ana Maria Pandolfo Feoli, Andréia da Silva Gustavo, Márcio Vinícius Fagundes Donadio

**Affiliations:** 1 Pontifícia Universidade Católica do Rio Grande do Sul Porto AlegreRS Brazil Pontifícia Universidade Católica do Rio Grande do Sul , Porto Alegre , RS , Brazil .

**Keywords:** Obesity, Overweight, Adolescent, Motivational interviewing, Exercise, Motivation

## Abstract

**Objective:**

To evaluate the effect of an interdisciplinary intervention with a motivational approach on exercise capacity and usual physical activity levels in overweight and obese adolescents.

**Methods:**

This is a randomized, controlled clinical trial with single blinding of subjects. Adolescents aged 15 to 18 years with overweight and obesity (body mass index ≥ 85 percentile) were included. The adolescents were randomized into two groups: interdisciplinary intervention or control − traditional approach aiming at lifestyle modifications. The initial evaluations were carried out, including the cardiopulmonary exercise test and the physical activity level measurement by using the International Physical Activity Questionnaire and a pedometer. The evaluations were performed in two moments: time zero (time of inclusion in the study) and after 3 months (end of intervention). There were 12 sessions with weekly meetings.

**Results:**

A total of 37 participants were included, 19 in the Intervention Group. There were no significant differences in the baseline demographic, anthropometric and physical activity characteristics between groups, with mean age of 17.3±1.0 years in the Control Group, and 16.8±0.9 years in the Intervention Group (p=0.14). The motivational intervention did not cause significant differences (p>0.05) in the comparison of the variables of exercise capacity and usual physical activity (questionnaire and pedometer) between groups.

**Conclusion:**

The intervention with a motivational approach did not alter exercise capacity and levels of usual physical activity in overweight and obese adolescents.

**Clinical Trial Registry:** NCT02455973 and REBEC: RBR-234nb5.

## INTRODUCTION

Obesity is one of the most concerning public health problems of the 21^st^ century, having as main causes genetic and non-genetic factors.^( 1 )^ In children and adolescents, growing concern is generated due to the great risk of association with diseases that modify blood pressure, triglycerides and cholesterol levels.^( 2 , 3 )^ In parallel, we note an increase in sedentary behavior in the pediatric age range as a result of increasing use of new technologies and lack of incentives for physical activity in schools and homes.^( 4 , 5 )^

In this sense, strategies for lifestyle changes become important by means of regular physical activity and adherence to a healthier diet, contributing towards the prevention and treatment of some cardiovascular risk factors.^( 6 )^ It is already known that physical activity in adolescents decreases the risk of cardiovascular diseases in adults, and contributes towards the prevention of type 2 *diabetes mellitus* and cardiovascular diseases, even without reducing body weight.^( 7 )^ Thus, the search for alternatives that contribute towards a change in lifestyle have been increasingly considered.^( 8 )^

Studies have investigated different models that might modify health-related behaviors, including the Transtheoretical Model (TTM) of change,^( 9 - 11 )^ which aims to understand, measure, and intervene in behavioral change.^( 10 )^ It is divided into stages that represent when change occurs and the degree of motivation to perform it.^( 12 )^

One of the factors that may interfere in the performance of treatment is motivation.^( 13 )^ Therefore, the use of the motivational interview (MI), along with the TTM, can contribute in this regard, considering that it deals with a type of care that evokes in the patients the motivation to make behavioral changes in the interest in one’s own health.^( 14 )^

Allied with TTM, the MI constitutes an alternative therapy to approach behavior change, which stimulates a constructive relationship between the healthcare professional and the patient, seeking to provide better treatment results. Considering that adolescents tend to spend a lot of time in sedentary activities, and that regular practice of physical activity reduces the risk of chronic diseases, the study of an interdisciplinary intervention with a motivational approach is justified for overweight and obese adolescents.

## OBJECTIVE

To evaluate the effect of an interdisciplinary intervention with a motivational approach on exercise capacity and the level of daily physical activity in adolescents who are overweight and obese.

## METHODS

This is a randomized, controlled, single blind study, recorded in both, REBEC (RBR-234nb5) and Clinical Trials (NCT02455973), which followed the CONSORT recommendations.^( 15 )^The study included adolescents aged between 15 and 18 years, and body mass index (BMI) consistent with overweight or obesity (≥85^th^ percentile). Excluded individuals were those who presented with any absolute contraindication (musculoskeletal, neurological, vascular, pulmonary, or cardiac problems) for physical activity, diagnosis of severe psychiatric disorders, and/or presence of significant cognitive damage, gestation, diagnosis of type 1 *diabetes mellitus* , and difficulty in returning for check-ups. The study was approved by the Research Ethics Committee of the *Pontifícia Universidade Católica do Rio Grande do Sul* (PUCRS), opinion number: 2.012.173, CAAE: 65233516.5.0000.5336. All parents and/or legal guardians signed the Informed Consent Form (ICF), and the adolescents signed the Assent Form (AF).

Recruitment of participants occurred by digital media, television, and radio. After showing initial interest, the adolescents were contacted by telephone to participate in a triage. At this point, they were explained the objectives of the study, and the inclusion and exclusion criteria were evaluated by means of anthropometric assessment and clinical data. After verifying eligibility criteria and obtaining the written consent from the parents and/or legal guardians, the adolescents were randomized through the Research Randomizer software, version 4.0, to participate in the traditional approach (Control Group − CG) or interdisciplinary intervention (Intervention Group − IG), seeking to modify lifestyle. One member of the research team was responsible for the blinding attribution of each participant and allocating them to one of the two experimental groups.

Posteriorly, primary assessments were scheduled for all participants with the research team. The evaluations included the cardiopulmonary exercise test (CPET) and checking the level of daily physical activity, by means of a questionnaire and of an objective measurement (pedometer). Evaluations were made at the Pediatric Physical Activity Laboratory at two times: time zero (moment of study inclusion) and after 3 months of intervention (end of the intervention).

The interventions were performed as per the protocol published in the International Journal of Clinical Trials,^( 16 )^in 12 sessions for each group, briefly described below.

In the CG, the focus of the sessions was the development of skills, by means of educative actions in health, using transmission pedagogy, in which the adolescents only received instructions about what they should do to modify eating habits and physical activity engagement. The group was led by a team composed of one member from each area, nursing, physical therapy, nutrition, and psychology, e followed a lecture schedule that covered the cardiovascular risk factors and their prevention. The meetings were held weekly, lasting for one hour, during 3 months.

In the IG, sessions were focused on the development of skills, by educative actions in health that afforded the development of autonomy and empowerment for the change in eating habits and physical activity, based on interdisciplinary motivational strategies. Meetings were held in the presence of a member of the nursing, physical therapy, nutrition, and psychology teams, lasting for one hour, during 3 months. In the first 60 minutes, health themes were addressed using the technical bases of the MI. At the end, 30 minutes were used so that the participants, together with the research team, could have an experience of the practice of guided physical activity, using the interactive videogame, Xbox. Inclusion of this moment of exercise sought to motivate the participant to include other sessions during the week. Intervention in this group allowed greater interaction between the health professionals and the adolescents, and encouraged active participation of the young people.

In both groups, meetings were held only with the parents or legal guardians of the adolescents. These meetings happened at the start and end of the intervention, with the objective of involving the family in the process to change lifestyle of the adolescents. More information about the sessions can be found in the study protocol.^( 16 )^

The primary endpoint of the study was the peak oxygen consumption (VO_2_peak) and the secondary endpoint was the level of daily physical activity. Additionally, a questionnaire was used that addressed sociodemographic data (age, sex, social class, skin color), and an anthropometric evaluation was made.

Measurements of body weight and height were triplicated or until obtaining two identical values. Body weight was measured with the individuals in orthostatic position, barefoot, with a minimum amount of clothes, using a previously calibrated digital scales (G-Tech, Glass 1 FW, Rio de Janeiro, Brazil) with 100g precision. Height was measured by means of a portable stadiometer (Altura Exata, TBW, São Paulo, Brazil) with precision of one millimeter, in orthostasis, with feet bare and parallel, and arms extended along the body.

The CPET was done as per recommendations of the American Thoracic Society (ATS) and American College of Chest Physicians (CHEST).^( 17 )^ All the tests were performed at room temperature between 22 and 24°C, and relative air humidity of around 60%. The assessment was done on a computerized system (Aerograph, AeroSport^®^, USA), coupled with a gas analyzer (VO_2000_, MedGraphics^®^, USA), and utilizing a treadmill (KT-10400, Inbramed^®^, Brazil). The variables collected during the test included VO_2_peak, the production of carbon dioxide (VCO_2_), minute ventilation (V_E_), respiratory exchange ratio (RER), ventilatory equivalents for oxygen consumption (V_E_/VO_2_) and for production of carbon dioxide (V_E_/VCO_2_), and the maximum heart rate (HRmax). The test was done with the ramp protocol, adapted according to a prior study.^( 18 )^ Participants were instructed to walk for 2 minutes to adapt to the treadmill, with a velocity of 3km/hour and no inclination. After this, there were speed increments of 0.5km/hour at every minute, with inclination set at 3% until the end of the test. All subjects were encouraged to keep up the rhythm until exhaustion or the appearance of limiting signs and/or symptoms (dyspnea, leg pain, and/or dizziness). To consider the test as maximal, at least three of the following criteria were to be observed: exhaustion of the participant, RER >1.0, HRmax>85% of the estimated HR (formula: 220 - age), and the presence of a plateau in VO_2_.^( 19 , 20 )^ At beginning and end of the test, data were collected regarding HR and peripheral oxygen saturation (SpO_2_), by means of a pulse oximeter (Nonin^®^, Minneapolis, USA), blood pressure (sphygmomanometer BIC, Itupeva, Brazil), and subjective perception of dyspnea and fatigue of the lower limbs evaluated by the modified Borg scale.^( 21 )^ Heart rate and SpO_2_ were monitored during the entire CPET protocol.

To rate the level of the adolescents’ daily physical activity level, the short version of the International Physical Activity Questionnaire (IPAQ)^( 22 )^ was used. This validated questionnaire covers questions about the previous 7 days, evaluating the frequency and time spent on moderate and vigorous activities and walks, besides the time spent in seated activities. The endpoint of this instrument is based on the time spent (minutes) in each one of the activities; those who engaged in at least 150 minutes of moderate intensity physical activity were classified as active.^( 22 )^ With the purpose of evaluating the activities carried out in everyday life in an objective manner, the adolescents also used, at the start and end of the intervention, a pedometer (DIGI-WALWER Electronic Pedometer SW700/701). Thus, during 7 days of use, the device recorded the quantity of steps, the distance covered, and the calories burned by the individuals. Participants were instructed to use the device on their waist for 7 days, on the right side, next to the iliac crest, taking it off only for sleeping, bathing, or any contact physical exercise. These instruments were given out along with oral and written instructions about them. Later, the devices were retrieved for data analysis and comparison with the data obtained from the questionnaires.

Calculation of the sample size was based on the variability of the VO_2_peak, since this is the primary endpoint variable. Data from evaluation of the adolescents at the same laboratory were also used. Considering a mean of VO_2_ of 30.55mL.kg^-1^.min^-1^, a standard deviation of 4.09mL.kg^-1^.min^-1^, a minimal difference to be detected of 2.7mL.kg^-1^.min^-1^, and power of 80% (i-β), and assuming a significance level of 5% (α=0.05), the sample size was estimated at 17 participants per group.

For statistical purposes, the normality of the data was assessed by means of the Shapiro-Wilk test. Continuous variables, with symmetric distribution, were presented as mean and standard deviation, while asymmetric data were shown as median and interquartile range. Categorical data were expressed as absolute and relative frequency. The independent Student’s *t* test or the Mann Whitney test were used to compare the characteristics between the groups, according to the symmetry of the data. Comparisons between the times before and after the intervention in both groups were done by paired Student’s *t* test or by Wilcoxon test. All the analyses and the data processing were performed with the (SPSS) version 18.0 (SPSS Inc., USA). The adopted significance level was p≤0.05.

## RESULTS

**Figure 1:**
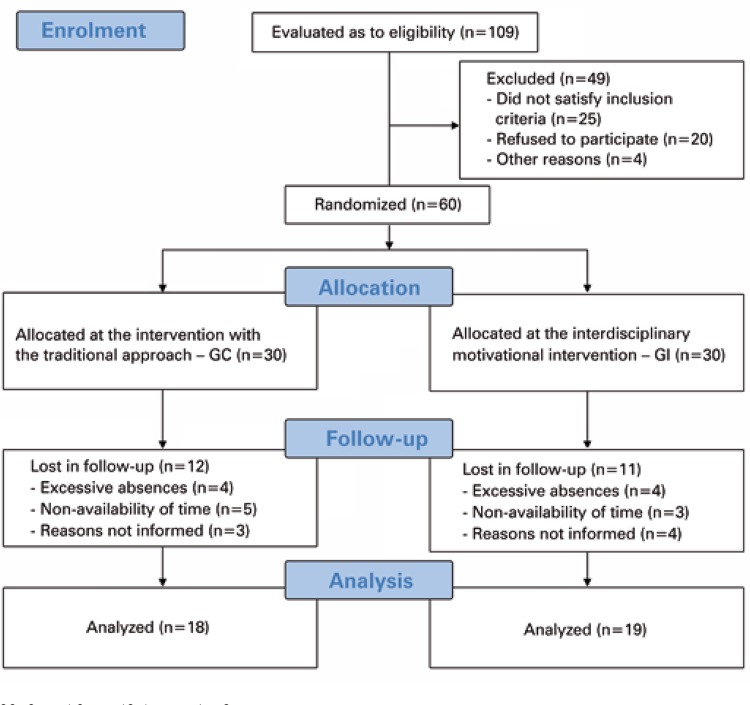
CG: Control Group; IG: Intervention Group. . Flowchart of study participants

From a total of 30 subjects allocated to each group, 18 adolescents completed the study in CG, and 19 in IG. figure 1 presents the flowchart of the study with data on subject selection and reasons for exclusion.

The mean age of the participants was 17.3±1.0 years in the CG and 16.8±0.9 years in the IG, with a predominance of the female sex in both groups. The mean BMI (Z score) was about 2, in which 17 (45.95%) adolescents were classified as overweight and 20 (54.05%) as obese. The median of time spent in moderate and vigorous physical activity was less than 300 minutes/week, and the time spent sitting/lying down was more than 800 minutes/week, indicating a behavior of inactivity. The mean of steps a day was 5,148.1±1,691.2 in the CG and 4,009.2±1,381.5 in the IG. No significant baseline differences were found (p *>* 0.05) in the comparison of demographic, anthropometric, and daily physical activity characteristics between CG and IG ( Table 1 ).

**Table 1:** Comparison of demographic, anthropometric, and levels of daily physical activity characteristics between the Control and Intervention Groups at baseline

Evaluated variables	CG (n=18)	IG (n=19)	p value
Demographic characteristics			
Age, years	17.3±1.0	16.8±0.9	0.139
Female sex	14 (77.8)	14 (73.7)	1.000
Anthropometric			
Body mass, kg	94.1±24.4	96.6±17.9	0.728
Height, cm	163.2±8.4	167.0±9.5	0.214
BMI, absolute	34.9±6.1	34.4±4.4	0.805
BMI, Z score	2.0±0.5	2.1±0.4	0.829
BMI, percentile	96.5±3.7	97.5±2.4	0.377
Levels of physical activity			
Questionnaire, minutes/week			
Vigorous activities	70.0 (0.0-141.2)	89.5 (60.0-300.0)	0.233
Moderate activities	125.0 (60.0-240.0)	90.0 (60.0-150.0)	0.558
Walking	87.4 (45.0-232.5)	75.0 (50.0-100.0)	0.461
Sitting/lying down	870.0 (630.0-1,680.0)	1,140.0 (720.0-1,320.0)	0.893
Pedometer, total steps/day	5,148.1±1,691.2	4,009.2±1,381.5	0.100

Results expressed as mean±standard deviation, n (%), or median, and interquartile range.

CG: Control Group; IG: Intervention Group; BMI: body mass index.

Table 2 shows the comparison of the variables of the CPET between CG and IG at baseline. The mean HR in CG was 186.0±10.1bpm, and in IG, it was 187.7±10.3bpm. The mean VO_2_ at peak of exercise in CG was 25.4±5.0mL.kg^-1^.min^-1^, and in IG, 27.9±5.6mL.kg^-1^.min^-1^, while the VO_2_ at the anaerobic threshold (AT) in CG was 21.9±4.9mL.kg^-1^.min^-1^, and in the IG, 22.1±5.9mL.kg^-1^.min^-1^. Subjective levels of lower limb fatigue/dyspnea were lower than 6 points in both groups. All cardiovascular, metabolic, ventilatory, and subjective variables at the peak of exercise were similar in both groups, except the V_E_, which was significantly higher (p=0.001) in IG as compared to the CG.

**Table 2:** Comparison of variables of the cardiopulmonary exercise test between the Control and Intervention Groups, at baseline

Evaluated variables	CG (n=18)	IG (n=19)	p value
Cardiovascular			
HR, bpm	186.0±10.1	187.7±10.3	0.608
VO_2_/HR, L/beats	12.5±12.6	14.3±3.9	0.096
SBP, mmHg	148.5±19.9	150.8±21.1	0.775
DBP, mmHg	79.2±9.5	75.0±13.1	0.364
SpO_2_, %	95.3±3.4	96.3±1.8	0.277
Metabolic			
RER	1.1±0.1	1.2±0.1	0.204
V_E_/VO_2_	21.9±2.8	21.6±2.2	0.700
V_E_/VCO_2_	21.2±2.8	21.1±2.1	0.868
Ventilatory			
V_E_, L.min^-1^	59.3±9.0	74.6±15.6	0.001
VO_2_peak, L.min^-1^	2.3±0.4	2.7±0.8	0.068
VO_2_peak, mL.kg^-1^.min^-1^	25.4±5.0	27.9±5.6	0.165
VO_2_ at AT, L.min^-1^	2.0±0.5	2.1±0.8	0.550
VO_2_ at AT, mL.kg^-1^.min^-1^	21.9±4.9	22.1±5.9	0.921
Subjective			
Borg for leg fatigue, score	4.9±2.0	5.4±2.6	0.585
Borg for dyspnea, score	4.3±2.0	5.1±2.4	0.267

Results expressed as mean ± standard deviation.

CG: Control Group; IG: Intervention Group; HR: heart rate; VO_2_: oxygen consumption; SBP: systolic blood pressure; DBP: diastolic blood pressure; SpO_2_: oxygen saturation; RER: respiratory exchange ratio; V_E_/VO_2_: ventilatory equivalent for oxygen consumption; V_E_/VCO_2_: ventilatory equivalent for carbon dioxide production; V_E_: minute ventilation; AT: anaerobic threshold.

Comparisons of the primary variables of the CPET and of the level of daily physical activity before and after the end of the intervention in each group are presented on table 3 . Control Group presented with a significant increase in V_E_ and V_E_/VCO_2_ at the end of the intervention. In IG, there was a significant decrease in V_E_ and increase in the walking time evaluated by the physical activity questionnaire.

**Table 3:** Comparison of the cardiopulmonary exercise test and of the levels of daily physical activity before and after the end of the intervention

Evaluated variables	Initial CG	Final CG	p value	Initial IG	Final IG	p value
CPET						
V_E_, L.min^-1^	59.4±9.0	68.3±14.4	0.024	74.6±15.6	64.9±18.5	0.005
VO_2_peak, mL.kg^-1^.min^-1^	25.4±5.1	27.6±4.9	0.071	27.9±5.6	25.4±6.3	0.061
VO_2_ at AT_,_ mL.kg^-1^.min^-1^	21.9±4.9	22.8±6.3	0.595	22.1±5.9	20.5±6.9	0.376
V_E_/VO_2_	21.9±2.9	22.6±2.8	0.073	21.6±2.2	22.6±1.9	0.088
V_E_/VCO_2_	21.3±2.8	22.2±2.8	0.007	21.1±2.1	22.2±1.9	0.066
Levels of physical activity						
Questionnaire, minutes/without						
Vigorous activities	70.0 (0.0-141.2)	127.5 (64.9-292.5)	0.132	89.5 (60.0-300.0)	120.0 (0.0-240.0)	0.218
Moderate activities	125.0 (60.0-240.0)	150.9 (7.5-262.5)	1.000	90.0 (60.0-150.0)	140.0 (60.0-270)	0.407
Walking	87.4 (45.0-232.5)	95.0 (45.0-232.5)	0.737	75.0 (50.0-100.0)	180.0 (79.0-300.0)	0.003
Sitting/lying down	870.0 (630.0-1,680.0)	870 (547.5-1,275.0)	0.111	1,140.0 (720.0-1,320.0)	720.0 (600-1.200)	0.074
Pedometer, steps/day	4,710.1±1,663.3	3,499.6±1,884.8	0.200	4,290.2±1,232.9	4,098.5±1,444.9	0.713

CG: Control Group; IG: Intervention Group; CPET: cardiopulmonary exercise test; V_E_: minute ventilation; VO_2_: oxygen consumption; V_E_/VO_2_: ventilatory equivalent for oxygen consumption; V_E_/VCO_2_: ventilatory equivalent for carbon dioxide production.

When the effects of the 3 months of intervention were evaluated, there were no significant modifications between CG and IG as to the exercise capacity rated by CPET (VO_2_peak, VO_2_ at AT, and ventilatory equivalents), with the exception of V_E_ (L.min^-1^), in which the variation was 4.9±20.7 in CG, and -5.9±10.3 in IG (p=0.048). Similarly, when the effect of the intervention was tested in the comparisons of variations in levels of daily physical activity obtained by the self-reported questionnaire, and by the use of pedometers, once again, no significant changes were found between CG and IG. The data are shown on table 4 .

**Table 4:** Comparison of the before and after diferences (delta) in the cardiopulmonar exercise test and levels of daily physical activity between the Control and Intervention Groups

Evaluated variables	CG	GI	Difference between means	p value	95%CI
CPET					
ΔV_E_, L.min^-1^	4.9±20.7	-5.9±10.3	-10.9	0.048	-21.7-0.1
ΔVO_2_ peak, mL.kg^-1^.min^-1^	0.7±6.9	-0.9±4.2	-1.7	0.376	-1.7-1.9
ΔVO_2_ at AT_,_ mL.kg^-1^.min^-1^	-1.1±7.9	0.2±6.9	1.4	0.582	-3.6-6.3
ΔV_E_/VO_2_	1.2±1.7	0.4±2.1	-0.7	0.231	-2.0-0.5
ΔV_E_/VCO_2_	1.3±1.5	0.7±2.2	-0.7	0.280	-1.9-0.6
Levels of physical activity					
Questionnaire, minutes/week					
Δ vigorous activities	55.6±221.1	-35.6±227.8	-91.1	0.239	-245.6-63.4
Δ moderate activities	7.8±123.7	92.4±180.3	84.7	0.113	-21.2-190.5
Δ walking	86.5±247.5	109.2±151.0	22.7	0.738	-114.5-159.9
Δ sitting/lying down	-204.2±482.2	-157.6±359.6	46.5	0.740	-236.3-329.4
Pedometer, steps/day					
Δ total number of steps	-1,210.5±2,237.9	-191.7±1,598.4	1,018.7	0.289	-956.6-2,993.9

Results expressed as ± standard deviation.

CG: Control Group; IG: Intervention Group; 95%CI: 95% confidence interval; CPET: cardiopulmonary exercise test; Δ: variation of the results (post - pre-intervention); V_E_: minute ventilation; VO_2_: oxygen consumption; V_E_/VO_2_: ventilatory equivalent for oxygen consumption; V_E_/VCO_2_: ventilatory equivalent for carbon dioxide production.

## DISCUSSION

Transtheoretical Model is a tool based on behavioral changes, and is composed of stages that allow a reflection on behavior, actions to be taken, and the moment to act.^( 23 )^The findings of the present study showed no significant change in the capacity for exercise evaluated by the CPET, including the VO_2_peak, in overweight and obese adolescents. Additionally, the levels of habitual physical activity, evaluated both by the questionnaire, and by pedometers, remained similar during the period studied.

Obesity is an important public health problem, since its prevalence has been increasing over the last decades, and has affected developing countries.^( 24 )^ Studies have shown the relation between the increase in BMI in the population with the growth in incidence of chronic diseases, such as coronary artery diseases, type 2 *diabetes mellitus* , and a decrease in disease-free time of life.^( 25 )^ One of the forms of treating obesity is based on lifestyle modification, and its early implementation is recommended as the first line approach for reducing cardiometabolic risk.^( 26 )^ Two meta-analyses of intervention studies in lifestyle for the pediatric age group showed that a modification in diet, associated with increased physical activity, can decrease weight in addition to improving risk factors, such as dyslipidemia and hypertension.^( 8 , 27 )^ Is it important to point out that the mean time of the interventions was more than 3 months in 65% of studies included.^( 8 )^ Although our study tested an intervention that sought to change the lifestyle of adolescents by means of the use of the TTM, there were no effects on the BMI and habitual physical activity. It is possible that the period of only 3 months of intervention, with meetings once a week, were not sufficient to reach the desired goals. Another possible hypothesis is that the adolescents were in a pre-contemplation stage, since they often were taken by their parents or guardians.

The TTM was initially created in the 1980’s for use with individuals who smoked_,_^( 9 )^ and presented a structure that sought to understand, measure, and intervene in behavioral change.^( 10 )^ Evidence has shown that this model may be considered a promising instrument in helping understanding health-related behavior change.^( 11 )^ In a study conducted with obese children evaluating the effects of 6 months of exercise intervention, using the TTM, there was maintenance of the blood sugar levels and of the BMI in the IG, besides an increase in control.^( 28 )^ Another study reported that physical activity interventions, based on TTM, were effective in the promotion of the physical activity levels among young adults.^( 29 )^Our findings demonstrated that there were no significant modifications in the variables studied after an intervention with the TTM, including the capacity for exercise and the levels of daily physical activity. Nevertheless, when comparing the data before and after the study period, there was an increase in V_E_ not associated with increments in CO_2_ elimination in the CG, which could indicate lower ventilatory efficiency. Although these studies^( 28 , 29 )^ used the same TTM model, the interventions were distinct in their essence, in addition to the time of intervention in adolescents and the themes covered during the sessions, hindering comparison with the results obtained in the present study.

The pattern of physical activity of adolescents can determine part of the levels of activity when they are adults.^( 30 )^ Adolescents have the tendency to spend a lot of time in low intensity activities, such as engaged in videogames, using the computer, and watching television, which has contributed to weight gain.^( 31 , 32 )^ On the other hand, evidence shows that adolescents who engage in physical activity have a lower risk of chronic diseases, including obesity.^( 33 )^ In the present study, despite not having found effects of the intervention in comparison to the CG in levels of daily physical activity, when comparing the data before and after the end of the intervention, the IG presented with a significant increase in the walking time. A systematic review demonstrated that the studies utilizing the TTM with the objective of weight loss in adults showed low methodology quality, limiting the conclusion that this intervention could lead to better habits in eating and physical activity.^( 34 )^ Nonetheless, in a study recently done with obese women, the use of the TTM for 3 months induced a greater loss of weight in the IG when compared to the control group.^( 35 )^ It is possible that the application of this model of intervention in adolescents could be an additional limiting factor when compared to the adult age range, which may have also contributed to explaining our results. A meta-analysis conducted in 2006 identified 64 obesity prevention programs for children and adolescents, and only 21% of them produced significant results.^( 36 )^

This study has some limitations, including the low frequency of weekly meetings (once a week) with the adolescents, as well as the need for greater involvement of the family throughout the intervention with the TTM, since only two meetings were held with the parents and/or guardians during the study. However, the choice of this weekly frequency was made due to the difficulty of gathering the participants on more days of the week, due to commitments to school activities, and because it is a transition phase of activities and social commitments. Additionally, we point out the objective of the study was not to hold daily sessions with closed rules, but rather to stimulate the change process.

## CONCLUSION

Our data did not show modifications in the variables of exercise capacity evaluated by the maximum effort test, including peak oxygen consumption in overweight and obese young people. Added to this, the levels of daily physical activity remained at similar levels in the sample during the study period. Evaluation of an intervention for a longer period of time and/or with greater frequency can be the object of future studies covering the theme of obesity in adolescents.
