# Supplementary material for: Effect of an interdisciplinary intervention with motivational approach on exercise capacity in obese adolescents: a randomized controlled clinical trial
Source: Einstein (Sao Paulo). 2020 May 13;18:eAO5268. doi: 10.31744/einstein_journal/2020AO5268 (PMC7233282; doi:10.31744/einstein_journal/2020AO5268)
Supplement: Translation

# Translation: Efeito de intervenção interdisciplinar com abordagem motivacional na capacidade de exercício em adolescentes obesos: ensaio clínico controlado randomizado

**Authors:** Letiane Bueno Zanatta, João Paulo Heinzmann-Filho, Fernanda Maria Vendrusculo, Natália Evangelista Campos, Margareth da Silva Oliveira, Ana Maria Pandolfo Feoli, Andréia da Silva Gustavo, Márcio Vinícius Fagundes Donadio

**Affiliations:** 1 Brasil Pontifícia Universidade Católica do Rio Grande do Sul, Porto Alegre, RS, Brasil .

**Keywords:** Obesidade, Sobrepeso, Adolescente, Entrevista motivacional, Exercício físico, Motivação

## Abstract

**Objetivo:**

Avaliar o efeito de uma intervenção interdisciplinar com abordagem motivacional na capacidade de exercício e no nível de atividade física habitual em adolescentes com sobrepeso e obesidade.

**Métodos:**

Trata-se de ensaio clínico controlado, randomizado, com cegamento único dos indivíduos. Foram incluídos adolescentes com idade entre 15 e 18 anos, com sobrepeso e obesidade (índice de massa corporal ≥ percentil 85). Os adolescentes foram randomizados em dois grupos: intervenção interdisciplinar motivacional ou controle − abordagem tradicional, visando à modificação do estilo de vida. Foram realizadas as avaliações iniciais incluindo o teste de exercício cardiopulmonar e a aferição do nível de atividade física por meio do *International Physical Activity Questionnaire* e do pedômetro. As avaliações foram realizadas em dois momentos, no tempo zero (inclusão no estudo) e após 3 meses (término da intervenção). Foram realizadas 12 sessões com encontros semanais.

**Resultados:**

Foram incluídos 37 participantes, sendo 19 no Grupo Intervenção. Não houve diferenças significativas nos dados basais de características demográficas, antropométricas e de atividade física entre os grupos, e a média de idade foi de 17,3±1,0 anos no Grupo Controle e 16,8±0,9 anos no Intervenção (p=0,14). A intervenção motivacional não provocou diferenças significativas (p>0,05) na comparação das variáveis de capacidade de exercício e atividade física habitual (questionário e pedômetro) entre os grupos.

**Conclusão:**

A intervenção com abordagem motivacional não alterou a capacidade de exercício e os níveis de atividade física habitual em adolescentes com sobrepeso e obesidade.

**Clinical Trial Registry:** NCT02455973 and REBEC: RBR-234nb5.

## INTRODUÇÃO

A obesidade é um dos problemas de saúde pública mais preocupante do século XXI, tendo como principais fatores aspectos genéticos e não genéticos.^( 1 )^Em crianças e adolescentes, gera cada vez mais preocupação, devido ao grande risco de associação com doenças que alteram a pressão arterial e os níveis de triglicerídeos e colesterol.^( 2 , 3 )^Em paralelo, nota-se aumento no sedentarismo na faixa etária pediátrica, devido ao crescente uso de novas tecnologias e à falta de incentivo para a prática de atividade física nas escolas e domicílios.^( 4 , 5 )^

Neste sentido, tornam-se importantes as estratégias de mudança do estilo de vida, por meio da prática regular de atividade física e da adesão a uma dieta mais saudável, contribuindo para a prevenção e o tratamento de alguns dos fatores de risco cardiovascular.^( 6 )^Já se sabe que a prática de atividade física na adolescência diminui o risco de doenças cardiovasculares na idade adulta, além de contribuir para a prevenção da *diabetes mellitus* tipo 2 e de doenças cardiovasculares, mesmo sem redução de peso corporal.^( 7 )^Assim, a busca por alternativas que contribuam para a mudança do estilo de vida tem sido cada vez mais estudada.^( 8 )^

Estudos têm investigado diferentes modelos que possam modificar comportamentos de saúde, incluindo o Modelo Transteórico de Mudança Comportamental (MTT),^( 9 - 11 )^o qual visa compreender, medir e intervir na mudança de comportamento,^( 10 )^sendo dividido em estágios que representam quando a mudança ocorre e qual é seu grau de motivação para realizá-la.^( 12 )^

Um dos fatores que pode interferir no desempenho do tratamento é a motivação.^( 13 )^Assim, a utilização da entrevista motivacional (EM), em conjunto com o MTT, pode contribuir neste sentido, considerando que se trata de um tipo de atendimento que evoca dos pacientes as motivações para fazer mudanças comportamentais no interesse de sua própria saúde.^( 14 )^

A EM aliada ao MTT configura terapêutica alternativa para abordar a mudança de comportamento, que estimula uma relação construtiva entre profissional da saúde e paciente, visando propiciar melhores resultados ao tratamento. Considerando que adolescentes apresentam tendência a despender muito tempo em atividades sedentárias e que a prática regular de atividade física reduz o risco de doenças crônicas, justifica-se o estudo de uma intervenção interdisciplinar com abordagem motivacional para adolescentes com sobrepeso e obesidade.

## OBJETIVO

Avaliar o efeito de uma intervenção interdisciplinar com abordagem motivacional sobre a capacidade de exercício e o nível de atividade física diária em adolescentes com sobrepeso e obesidade.

## MÉTODOS

Trata-se de ensaio clínico controlado, randomizado, com cegamento único dos indivíduos, registrado em ambos REBEC (RBR-234nb5) e *Clinical Trials* (NCT02455973), que seguiu as recomendações CONSORT.^( 15 )^Foram incluídos adolescentes com idade entre 15 e 18 anos e índice de massa corporal (IMC) compatível com sobrepeso ou obesidade (≥ percentil 85). Foram excluídos os indivíduos que apresentassem alguma contraindicação absoluta para a prática de atividade física (problemas musculoesqueléticos, neurológicos, vasculares, pulmonares ou cardíacos), diagnóstico de transtornos psiquiátricos graves e/ou presença de prejuízos cognitivos significativos, gestação, diagnóstico de *diabetes mellitus* tipo 1 e dificuldade de retorno nas avaliações.

O recrutamento dos participantes ocorreu por mídia digital, televisão e rádio. Após o interesse inicial, os adolescentes foram contatados por telefone para participarem de uma triagem. Nesse momento, foram explicados os objetivos do estudo e avaliados os critérios de inclusão e exclusão, por meio da avaliação antropométrica e das informações referentes aos dados clínicos. Após a verificação dos critérios de elegibilidade e obtenção do consentimento escrito dos pais e/ou responsáveis, os adolescentes foram randomizados por meio do software *Research Randomizer* , versão 4.0, para participar do grupo com abordagem tradicional (Controle − GC) ou intervenção interdisciplinar (Intervenção − GI), visando à modificação do estilo de vida. Um membro da equipe de pesquisa foi responsável pela atribuição cega de cada participante a um dos dois grupos experimentais.

Posteriormente, foram agendadas as avaliações iniciais de todos os participantes com a equipe de pesquisa. As avaliações incluíram o teste de exercício cardiopulmonar (TECP) e a aferição do nível de atividade física habitual, por meio de um questionário e de uma medida objetiva (pedômetro). As avaliações foram realizadas no Laboratório de Atividade Física em Pediatria, em dois momentos para ambos os grupos: no tempo zero (momento da inclusão no estudo) e após 3 meses de intervenção (término da intervenção).

As intervenções foram realizadas conforme protocolo publicado no *International Journal of Clinical Trials* ,^( 16 )^em número de 12 sessões para cada grupo, descritas resumidamente a seguir.

No GC, o foco das sessões foi o desenvolvimento de habilidades, por meio de ações educativas em saúde, utilizando a pedagogia da transmissão, na qual os adolescentes receberam apenas orientação sobre o que eles deveriam fazer para modificar os hábitos alimentares e a prática de atividade física. O grupo foi guiado por uma equipe composta por um membro de enfermagem, fisioterapia, nutrição e psicologia, e seguiu um cronograma de palestras que abordava os fatores de risco cardiovascular e a prevenção dos mesmos. Os encontros foram semanais com duração de 1 hora, durante 3 meses.

No GI, o foco das sessões foi o desenvolvimento de habilidades, por meio de ações educativas em saúde que proporcionassem o desenvolvimento da autonomia e do empoderamento para a mudança do comportamento alimentar e da prática de atividade física, com base em estratégias motivacionais interdisciplinares. Os encontros foram realizados na presença de um membro da equipe de enfermagem, fisioterapia, nutrição e psicologia, com encontros semanais, com duração de 1 hora e 30 minutos, durante 3 meses. Nos primeiros 60 minutos, foram abordados temas em saúde, utilizando os fundamentos da técnica da EM. Ao final, foram utilizados 30 minutos para que os participantes, em conjunto com a equipe de pesquisa, tivessem uma experiência de prática de atividade física orientada, utilizando o videogame interativo Xbox. A inclusão desse momento de exercício visou motivar o participante a incluir outras sessões durante a semana. A intervenção nesse grupo permitiu maior interação entre os profissionais da saúde e os adolescentes, além de incentivar a participação ativa dos jovens.

Em ambos os grupos, foram realizados dois encontros somente com os pais ou responsáveis legais dos adolescentes. Esses encontros aconteceram no início e no final da intervenção, com o objetivo de envolver a família no processo de mudança do estilo de vida dos adolescentes. Mais informações sobre as sessões podem ser encontradas no protocolo do estudo.^( 16 )^

O desfecho primário do estudo foi o consumo de oxigênio de pico (VO_2_pico) e o desfecho secundário foi o nível de atividade física diária. Além disso, foi utilizado um questionário que contemplava dados sociodemográficos (idade, sexo, classe social e cor) e foi realizada avaliação antropométrica.

As aferições da massa corporal e da estatura foram realizadas em triplicata ou até a obtenção de dois valores idênticos. A massa corporal foi obtida com os indivíduos em posição ortostática, descalços, com o mínimo de roupa, por meio de uma balança digital (G-Tech, Glass 1 FW, Rio de Janeiro, Brasil) com precisão de 100g, previamente calibrada. A altura foi mensurada por meio de um estadiômetro portátil (AlturaExata, TBW, São Paulo, Brasil) com precisão de um milímetro, em ortostase, com os pés descalços em posição paralela e braços estendidos ao longo do corpo.

O TECP foi realizado de acordo com as recomendações da *American Thoracic Society* (ATS) e *American College of Chest Physicians* (CHEST).^( 17 )^Todos os exames foram realizados com temperatura da sala entre 22 e 24°C e umidade relativa do ar em torno de 60%. A avaliação foi realizada em um sistema computadorizado (Aerograph, AeroSport^®^, EUA), acoplado a um analisador de gases (VO_2000_, MedGraphics^®^, EUA) e utilizando uma esteira rolante (KT-10400, Inbramed^®^, Brasil). As variáveis coletadas durante o teste incluíram o consumo máximo de oxigênio (VO_2_pico), a produção de dióxido de carbônico (VCO_2_), a ventilação minuto (V_E_), o coeficiente de troca respiratória (RQ), os equivalentes ventilatórios para o consumo de oxigênio (V_E_/VO_2_) e para produção de dióxido de carbono (V_E_/VCO_2_) e a frequência cardíaca máxima (FCmáx). O teste foi realizado com protocolo de rampa, adaptado de acordo com estudo prévio.^( 18 )^Os participantes foram orientados caminhar por 2 minutos para se adaptar à esteira, com velocidade de 3km/hora e sem inclinação. Após, ocorreram incrementos na velocidade de 0,5km/hora a cada minuto, com inclinação fixa em 3% até a finalização do teste. Todos foram encorajados a manter o ritmo até a exaustão ou surgimento de sinais e/ou sintomas limitantes (dispneia, dor nas pernas e/ou tontura). Para se considerar o teste como máximo, pelo menos três dos seguintes critérios deveriam ser observados: exaustão do participante, coeficiente de troca respiratória >1,0, FCmáx >85% da FC estimada (fórmula: 220 - idade) e a presença de platô no VO_2_.^( 19 , 20 )^No início e no final do teste, foram coletados os dados de FC e saturação periférica de oxigênio (SpO_2_), por meio de um oxímetro de pulso (Nonin^®^, Mineápolis, EUA), pressão arterial (esfignomanômetro BIC, Itupeva, Brasil), e percepção subjetiva de dispneia e fadiga de membros inferiores, avaliadas pela escala modificada de Borg.^( 21 )^A FC e a SpO_2_foram monitoradas durante todo o protocolo do TECP.

Para avaliar o nível de atividade física diária dos adolescentes, foi utilizado o *International Physical Activity Questionnaire* (IPAQ)^( 22 )^na versão curta. Esse questionário validado aborda questões sobre os últimos 7 dias, avaliando a frequência e o tempo gasto em atividades moderadas, vigorosas e caminhadas, além do tempo em atividades sentadas. O desfecho desse instrumento se baseia no tempo gasto (minutos) em cada uma das atividades, sendo classificados como ativos os indivíduos que praticam pelo menos 150 minutos de atividade física de intensidade moderada.^( 22 )^Com o objetivo de avaliar as atividades realizadas no dia a dia de forma objetiva, os adolescentes também utilizaram, no início e final da intervenção, um pedômetro (DIGI-WALWER Eletronic Pedometer SW700/701). Assim, durante 7 dias de uso, o aparelho registrou a quantidade de passos, a distância percorrida e as calorias gastas pelos indivíduos. Os participantes foram orientados a usar o aparelho na cintura por 7 dias, do lado direito, próximo à crista-ilíaca, tirando apenas para dormir, tomar banho ou algum exercício físico de contato. Estes instrumentos foram entregues juntamente das instruções orais e escritas sobre os mesmos. Após, os aparelhos foram recolhidos para a análise dos dados e comparação com os dados obtidos pelos questionários.

O cálculo do tamanho de amostra baseou-se na variabilidade do VO_2_pico, por ser esta a principal variável de desfecho. Foram utilizados dados oriundos de avaliações de adolescentes do próprio laboratório. Considerando média de VO_2_de 30,55mL.kg^-1^.min^-1^, desvio padrão de 4,09mL.kg^-1^.min^-1^, diferença mínima a ser detectada de 2,7mL.kg^-1^.min^-1^e poder de 80% (i-β), e assumindo um nível de significância de 5% (α=0,05), estimou-se tamanho amostral de 17 participantes por grupo.

Para fins estatísticos, a normalidade dos dados foi avaliada por meio do teste de Shapiro-Wilk. As variáveis contínuas, com distribuição simétrica, foram apresentadas em média e desvio padrão, enquanto os dados assimétricos foram apresentados em mediana e intervalo interquartílico. Os dados categóricos foram expressos como frequência absoluta e relativa. O teste *t* de Student independente ou o teste de Mann Whitney foram utilizados para comparar as características entre os grupos, de acordo com a simetria dos dados. As comparações entre os momentos antes e após a intervenção em ambos os grupos foram realizadas pelo teste *t* de Student pareado ou pelo teste de Wilcoxon. Todas as análises e o processamento dos dados foram realizados com o programa (SPSS) versão 18,0 (SPSS Inc., EUA). O nível de significância adotado foi de p≤0,05.

O estudo foi aprovado pelo Comitê de Ética em Pesquisa da Pontifícia Universidade Católica do Rio Grande do Sul (PUCRS), número do parecer: 2.012.173, CAAE: 65233516.5.0000.5336, e todos os pais e/ou responsáveis assinaram o Termo de Consentimento Livre e Esclarecido (TCLE) e os adolescentes o Termo de Assentimento (TA).

## RESULTADOS

De um total de 30 sujeitos alocados em cada grupo, 18 adolescentes completaram o estudo no GC e 19 no GI. A figura 1 apresenta o fluxograma do estudo com dados da seleção dos sujeitos e os motivos de exclusão.

**Figura 1:** Fluxograma dos participantes do estudo GC: Grupo Controle; GI: Grupo Intervenção.

A média de idade dos participantes foi 17,3±1,0 anos no GC e 16,8±0,9 anos no GI, com predomínio do sexo feminino nos dois grupos. A média do IMC (Escore Z) foi cerca de 2, sendo 17 (45,95%) adolescentes classificados com sobrepeso e 20 (54,05%) com obesidade. A mediana do tempo gasto em atividade física moderada e vigorosa foi menor que 300 minutos/semana, e o tempo sentando/deitado esteve acima de 800 minutos/semana, indicando comportamento de inatividade. A média de passos por dia foi de 5.148,1±1.691,2 no GC e 4.009,2±1.381,5 no GI. Não foram encontradas diferenças basais significativas (p *>* 0,05) na comparação das características demográficas, antropométricas e de atividade física diária entre o GC e o GI ( Tabela 1 ).

**Tabela 1:** Comparação das características demográficas, antropométricas e do nível de atividade física diária entre os Grupo Controle e Intervenção no momento inicial do estudo

Variáveis avaliadas	GC (n=18)	GI (n=19)	Valor de p
Características demográficas			
Idade, anos	17,3±1,0	16,8±0,9	0,139
Sexo feminino	14 (77,8)	14 (73,7)	1,000
Antropometria			
Massa corporal, kg	94,1±24,4	96,6±17,9	0,728
Estatura, cm	163,2±8,4	167,0±9,5	0,214
IMC, absoluto	34,9±6,1	34,4±4,4	0,805
IMC, escore Z	2,0±0,5	2,1±0,4	0,829
IMC, percentil	96,5±3,7	97,5±2,4	0,377
Nível de atividade física			
Questionário, minutos/semana			
Atividades vigorosas	70,0 (0,0-141,2)	89,5 (60,0-300,0)	0,233
Atividade moderadas	125,0 (60,0-240,0)	90,0 (60,0-150,0)	0,558
Caminhada	87,4 (45,0-232,5)	75,0 (50,0-100,0)	0,461
Sentado/deitado	870,0 (630,0-1.680,0)	1.140,0 (720,0-1.320,0)	0,893
Pedômetro, total de passos/dia	5.148,1±1.691,2	4.009,2±1.381,5	0,100

Resultados expressos em média±desvio padrão, n (%) ou mediana e intervalo interquartil.

GC: Grupo Controle; GI: Grupo Intervenção; IMC: índice de massa corporal.

A tabela 2 apresenta a comparação das variáveis do TECP entre o GC e o GI no momento inicial do estudo. A média da FC no GC foi de 186,0±10,1bpm e, no GI, de 187,7±10,3bpm. A média do VO_2_no pico do exercício no GC foi de 25,4±5,0mL.kg^-1^.min^-1^e, no GI, 27,9±5,6mL.kg^-1^.min^-1^, enquanto o VO_2_no limiar anaeróbico (LA) no GC foi de 21,9±4,9mL.kg^-1^.min^-1^e, no GI, de 22,1±5,9mL.kg^-1^.min^-1^. Os níveis subjetivos de cansaço em membros inferiores/dispneia ficaram abaixo dos 6 pontos em ambos os grupos. Todas as variáveis cardiovasculares, metabólicas, ventilatórias e subjetivas no pico do exercício foram semelhantes em ambos os grupos, com exceção da V_E_, que foi significativamente maior (p=0,001) no GI em comparação ao GC.

**Tabela 2:** Comparação das variáveis do teste de exercício cardiopulmonar entre os Grupo Controle e Intervenção, no momento inicial do estudo

Variáveis avaliadas	GC (n=18)	GI (n=19)	Valor de p
Cardiovasculares			
FC, bpm	186,0±10,1	187,7±10,3	0,608
VO_2_/FC, L/batimentos	12,5±12,6	14,3±3,9	0,096
PAS, mmHg	148,5±19,9	150,8±21,1	0,775
PAD, mmHg	79,2±9,5	75,0±13,1	0,364
SpO_2_, %	95,3±3,4	96,3±1,8	0,277
Metabólicas			
RQ	1,1±0,1	1,2±0,1	0,204
V_E_/VO_2_	21,9±2,8	21,6±2,2	0,700
V_E_/VCO_2_	21,2±2,8	21,1±2,1	0,868
Ventilatórias			
V_E_, L.min^-1^	59,3±9,0	74,6±15,6	0,001
VO_2_pico, L.min^-1^	2,3±0,4	2,7±0,8	0,068
VO_2_pico, mL.kg^-1^.min^-1^	25,4±5,0	27,9±5,6	0,165
VO_2_no LA, L.min^-1^	2,0±0,5	2,1±0,8	0,550
VO_2_no LA, mL.kg^-1^.min^-1^	21,9±4,9	22,1±5,9	0,921
Subjetivas			
Borg cansaço pernas, pontuação	4,9±2,0	5,4±2,6	0,585
Borg dispneia, pontuação	4,3±2,0	5,1±2,4	0,267

Resultados expressos como média±desvio padrão.

GC: Grupo Controle; GI: Grupo Intervenção; FC: frequência cardíaca; VO_2_: consumo de oxigênio; PAS: pressão arterial sistólica; PAD: pressão arterial diastólica; SpO_2_: saturação de oxigênio; RQ: coeficiente de troca respiratória; V_E_/VO_2_: equivalente ventilatório para o consumo de oxigênio; V_E_/VCO_2_: equivalente ventilatório para a produção de dióxido de carbono; V_E_: ventilação minuto; LA: limiar anaeróbico.

As comparações das principais variáveis do TECP e do nível de atividade física diária antes e após o final da intervenção em cada grupo são apresentadas na tabela 3 . O GC apresentou aumento significativo na V_E_e no V_E_/VCO_2_ao término da intervenção. Já no GI, ocorreram diminuição significativa na V_E_e aumento no tempo de caminhada avaliado pelo questionário de atividade física.

**Tabela 3:** Comparação do teste de exercício cardiopulmonar e do nível de atividade física diária antes e após o final da intervenção

Variáveis avaliadas	GC inicial	GC final	Valor de p	GI inicial	GI final	Valor de p
TECP						
V_E_, L.min^-1^	59,4±9,0	68,3±14,4	0,024	74,6±15,6	64,9±18,5	0,005
VO_2_pico, mL.kg^-1^.min^-1^	25,4±5,1	27,6±4,9	0,071	27,9±5,6	25,4±6,3	0,061
VO_2_no LA_,_mL.kg^-1^.min^-1^	21,9±4,9	22,8±6,3	0,595	22,1±5,9	20,5±6,9	0,376
V_E_/VO_2_	21,9±2,9	22,6±2,8	0,073	21,6±2,2	22,6±1,9	0,088
V_E_/VCO_2_	21,3±2,8	22,2±2,8	0,007	21,1±2,1	22,2±1,9	0,066
Nível de atividade física						
Questionário, minutos/semana						
Atividades vigorosas	70,0 (0,0-141,2)	127,5 (64,9-292,5)	0,132	89,5 (60,0-300,0)	120,0 (0,0-240,0)	0,218
Atividade moderadas	125,0 (60,0-240,0)	150,9 (7,5-262,5)	1,000	90,0 (60,0-150,0)	140,0 (60,0-270)	0,407
Caminhada	87,4 (45,0-232,5)	95,0 (45,0-232,5)	0,737	75,0 (50,0-100,0)	180,0 (79,0-300,0)	0,003
Sentado/deitado	870,0 (630,0-1.680,0)	870 (547,5-1.275,0)	0,111	1.140,0 (720,0-1.320,0)	720,0 (600-1.200)	0,074
Pedômetro, passos/dia	4.710,1±1.663,3	3.499,6±1.884,8	0,200	4.290,2±1.232,9	4.098,5±1.444,9	0,713

C: Grupo Controle; GI: Grupo Intervenção; TECP: teste de exercício cardiopulmonar; V_E_: ventilação minuto; VO_2_: consumo de oxigênio; V_E_/VO_2_: equivalente ventilatório para o consumo de oxigênio; V_E_/VCO_2_: equivalente ventilatório para a produção de dióxido de carbono.

Quando foram avaliados os efeitos dos 3 meses de intervenção, não houve alterações significativas entre o GC e GI sobre a capacidade de exercício avaliada pelo TECP (VO_2_pico, VO_2_no LA e equivalentes ventilatórios), com exceção da V_E_(L.min^-1^), em que a variação foi de 4,9±20,7, no GC, e -5,9±10,3, no GI (p=0,048). Da mesma forma, quando se testou o efeito da intervenção nas comparações das variações dos níveis de atividade física diária obtidos pelo questionário autorrelatado e pelo uso dos pedômetros, novamente não foram encontradas alterações significativas entre o GC e o GI. Os dados são apresentados na tabela 4 .

**Tabela 4:** Comparação da variação antes e depois (delta) do teste de exercício cardiopulmonar e do nível de atividade física diária entre os Grupos Controle e Intervenção

Variáveis avaliadas	GC	GI	Diferença entre médias	Valor de p	IC95%
TECP					
ΔV_E_, L.min^-1^	4,9±20,7	-5,9±10,3	-10,9	0,048	-21,7-0,1
ΔVO_2_pico, mL.kg^-1^.min^-1^	0,7±6,9	-0,9±4,2	-1,7	0,376	-1,7-1,9
ΔVO_2_no LA, mL.kg^-1^.min^-1^	-1,1±7,9	0,2±6,9	1,4	0,582	-3,6-6,3
ΔVE/VO_2_	1,2±1,7	0,4±2,1	-0,7	0,231	-2,0-0,5
ΔVE/VCO_2_	1,3±1,5	0,7±2,2	-0,7	0,280	-1,9-0,6
Nível de atividade física					
Questionário, minutos/semana					
Δ atividades vigorosas	55,6±221,1	-35,6±227,8	-91,1	0,239	-245,6-63,4
Δ atividade moderadas	7,8±123,7	92,4±180,3	84,7	0,113	-21,2-190,5
Δ caminhada	86,5±247,5	109,2±151,0	22,7	0,738	-114,5-159,9
Δ sentado/deitado	-204,2±482,2	-157,6±359,6	46,5	0,740	-236,3-329,4
Pedômetro, passos/dia					
Δ total de passos	-1.210,5±2.237,9	-191,7±1.598,4	1.018,7	0,289	-956,6-2.993,9

Resultados expressos como média ± desvio padrão.

GC: Grupo Controle; GI: Grupo Intervenção; IC95%: intervalo de confiança de 95%; TECP: teste de exercício cardiopulmonar; Δ: variação dos resultados (pós - pré-intervenção); V_E_: ventilação minuto; VO_2_: consumo de oxigênio; V_E_/VO_2_: equivalente ventilatório para o consumo de oxigênio; V_E_/VCO_2_: equivalente ventilatório para produção de dióxido de carbono.

## DISCUSSÃO

O MTT é uma ferramenta baseada nas mudanças de comportamento, sendo composto por estágios que possibilitam uma reflexão sobre o comportamento, a atitude a ser tomada e o momento de agir.^( 23 )^Os achados do presente estudo não evidenciaram mudanças significativas na capacidade de exercício avaliada pelo TECP, incluindo o VO_2_pico, em adolescentes com sobrepeso e obesidade. Além disso, os níveis de atividade física habituais, avaliados tanto por meio de questionário, como de pedômetros, permaneceram semelhantes durante o período estudado.

A obesidade é um importante problema de saúde pública, já que, nas últimas décadas, sua prevalência vem aumentando e acometendo países em desenvolvimento.^( 24 )^Estudos têm mostrado a relação do aumento no IMC da população com o crescimento na incidência de doenças crônicas, como doenças coronarianas, *diabetes mellitus* tipo 2 e diminuição do tempo de vida livre de doenças.^( 25 )^Umas das formas de tratamento da obesidade baseia-se na modificação do estilo de vida, sendo sua implementação precoce recomendada como abordagem de primeira linha para reduzir o risco cardiometabólico.^( 26 )^Duas metanálises de estudos de intervenção em estilo de vida na faixa etária pediátrica demonstraram que uma modificação da dieta, associada ao aumento da prática de atividade física, pode, além de diminuir o peso, melhorar fatores de risco, como dislipidemia e hipertensão.^( 8 , 27 )^É importante ressaltar que o tempo médio das intervenções foi superior a 3 meses em 65% dos estudos incluídos.^( 8 )^Embora nosso estudo tenha testado uma intervenção que visava à modificação do estilo de vida em adolescentes, por meio da utilização do MTT, não houve efeitos sobre o IMC e a prática de atividade física habitual. É possível que o período de apenas 3 meses de intervenção, com encontros uma vez por semana, não tenha sido suficiente para atingir as mudanças desejadas. Outra hipótese possível é que os adolescentes estivessem em estágio de pré-contemplação, já que, muitas vezes, eram levados pelos pais ou responsáveis.

O MTT foi criado inicialmente na década de 1980 para utilização com indivíduos tabagistas_,_^( 9 )^apresentando estrutura que visa compreender, medir e intervir na mudança de comportamento.^( 10 )^Evidências demonstram que esse modelo pode ser considerado instrumento promissor no auxílio à compreensão da mudança comportamental relacionada à saúde.^( 11 )^Em estudo feito com crianças obesas avaliando os efeitos de 6 meses de intervenção de exercício, utilizando o MTT, houve manutenção dos níveis de açúcar no sangue e do IMC no GI e aumento no controle.^( 28 )^Outro estudo relatou que intervenções de atividade física com base no MTT foram eficazes na promoção dos níveis de atividade física entre adultos jovens.^( 29 )^Nossos achados demonstraram que não houve alterações significativas nas variáveis estudadas após a intervenção com o MTT, incluindo a capacidade de exercício e os níveis de atividade física diária. Apesar disso, ao comparar os dados antes e após o período do estudo, houve aumento da V_E_, que não foi associado a um incremento na eliminação de CO_2_, no GC, o que poderia indicar menor eficiência ventilatória. Embora esses estudos^( 28 , 29 )^tenham usado o mesmo modelo MTT, as intervenções foram distintas em sua essência, além do tempo de intervenção e dos temas abordados nas sessões, dificultando a comparação com os resultados obtidos no presente estudo.

O padrão de atividade física da adolescência pode determinar parte dos níveis de atividade na idade adulta.^( 30 )^Adolescentes têm tendência a despender muito tempo em atividades de baixa intensidade, como jogar videogame, usar computador e assistir televisão, o que tem contribuído para o ganho de peso.^( 31 , 32 )^Por outro lado, evidências mostram que adolescentes que praticam atividade física têm menor risco de doenças crônicas − dentre elas a obesidade.^( 33 )^No presente estudo, apesar de não termos encontrado efeitos da intervenção em comparação ao GC nos níveis de atividade física diária, ao compararmos os dados antes e após o final da intervenção, o GI apresentou aumento significativo no tempo de caminhada. Uma revisão sistemática demonstrou que os estudos utilizando o MTT visando à perda de peso em adultos apresentam qualidade metodológica baixa, limitando a conclusão de que essa intervenção poderia levar a melhores hábitos alimentares e de atividade física.^( 34 )^No entanto, recentemente, em estudo feito com mulheres obesas, a utilização do MTT por 3 meses induziu uma maior perda de peso no GI em comparação ao controle.^( 35 )^É possível que a aplicação deste modelo de intervenção em adolescentes seja fator limitador adicional quando comparado à faixa etária adulta, o que também poderia ter contribuído para explicar nossos resultados. Uma metanálise realizada em 2006 identificou 64 programas de prevenção da obesidade para crianças e adolescentes, dos quais somente 21% produziram efeitos significativos.^( 36 )^

Este estudo apresenta algumas limitações, incluindo a baixa frequência de encontros semanais (uma vez por semana) com os adolescentes, bem como a necessidade de um maior envolvimento da família ao longo da intervenção com o MTT, já que se realizaram apenas dois encontros com os pais e/ou responsáveis ao longo do estudo. No entanto, a escolha dessa frequência semanal se deu pela dificuldade de reunir os participantes em mais dias na semana, devido aos compromissos com atividades escolares e por ser uma fase de transição das atividades e compromissos sociais. Além disso, cabe ressaltar que o objetivo do estudo não era realizar sessões diárias com regras fechadas, e sim, estimular o processo da mudança.

## CONCLUSÃO

Nossos dados não evidenciaram alterações nas variáveis de capacidade de exercício avaliadas pelo teste de esforço máximo, incluindo o consumo de oxigênio de pico, em adolescentes com sobrepeso e obesidade. Somando-se a isso, os níveis de atividade física diária permaneceram semelhantes durante o período estudado na amostra. A avaliação de uma intervenção por um período maior de tempo e/ou com maior frequência pode ser objeto de futuros estudos abordando a obesidade na adolescência.
